# The associations of BMI with mean diffusivity of basal ganglia among young adults with mild obesity and without obesity

**DOI:** 10.1038/s41598-020-69438-5

**Published:** 2020-07-28

**Authors:** Hikarua Takeuchi, Yasuyuki Taki, Rui Nouchi, Ryoichi Yokoyama, Seishu Nakagawa, Kunio Iizuka, Kohei Sakaki, Tsuyoshi Araki, Takayuki Nozawa, Shigeyuki Ikeda, Susumu Yokota, Sugiko Hanawa, Daniele Magistro, Yuka Kotozaki, Yukako Sasaki, Kelssy H. dos S. Kawata, Ryuta Kawashima

**Affiliations:** 1 Division of Developmental Cognitive Neuroscience, Institute of Development, Aging and Cancer (IDAC), Tohoku University, 4-1 Seiryo-cho, Aoba-ku, Sendai, 980-8575 Japan; 2 Division of Medical Neuroimaging Analysis, Department of Community Medical Supports, Tohoku Medical Megabank Organization, Tohoku University, Sendai, Japan; 3 Department of Radiology and Nuclear Medicine, Institute of Development, Aging and Cancer, Tohoku University, Sendai, Japan; 4 Creative Interdisciplinary Research Division, Frontier Research Institute for Interdisciplinary Science, Tohoku University, Sendai, Japan; 5 Human and Social Response Research Division, International Research Institute of Disaster Science, Tohoku University, Sendai, Japan; 6 Department of Advanced Brain Science, Institute of Development, Aging and Cancer, Tohoku University, Sendai, Japan; 7 School of Medicine, Kobe University, Kobe, Japan; 8 Department of Human Brain Science, Institute of Development, Aging and Cancer, Tohoku University, Sendai, Japan; 9 Division of Psychiatry, Tohoku Medical and Pharmaceutical University, Sendai, Japan; 10 ADVANTAGE Risk Management Co., Ltd., Tokyo, Japan; 11 Collaborative Research Center for Happiness Co-Creation Society Through Intelligent Communications, Tokyo Institute of Technology, Tokyo, Japan; 12 Department of Ubiquitous Sensing, Institute of Development, Aging and Cancer, Tohoku University, Sendai, Japan; 13 National Centre for Sport and Exercise Medicine (NCSEM), The NIHR Leicester-Loughborough Diet, Lifestyle and Physical Activity Biomedical Research Unit, School of Sport, Exercise, and Health Sciences, Loughborough University, Loughborough, England; 14 Division of Clinical Research, Medical-Industry Translational Research Center, Fukushima Medical University School of Medicine, Fukushima, Japan; 15 Center for Evolutionary Cognitive Sciences, University of Tokyo, Tokyo, Japan

**Keywords:** Striatum, Predictive markers

## Abstract

Obesity causes a wide range of systemic diseases and is associated with mood and anxiety disorders. It is also associated with dopaminergic reward system function. However, the relationships between microstructural properties of the dopaminergic system and body mass index (BMI) have not been investigated. In this study, we investigated the associations of BMI with mean diffusivity (MD), diffusion tensor imaging measure in areas of the dopaminergic system (MDDS) in 435 healthy young adults with mild obesity and without obesity (BMI < 40). We detected the association between greater BMI and lower MD of the right globus pallidus and the right putamen. These results suggest that the property of the dopaminergic system is associated with BMI among young adults with mild obesity and without obesity.

## Introduction

In the modern age, obesity is increasingly common across the globe, and this has caused a substantial increase in a wide range of diseases and mortality^1^. Not only that, obesity is associated with mood and anxiety disorders, and these associations appear to be bidirectional^2^. Further, it has been pointed out that the brain reward system is involved in regulating food intake^3^, and obesity and addiction, as well as conditions of the diminished capacity for enjoying the reward, are suggested to have overlapping neural mechanisms, according to multiple reviews^4^, and these conditions involve addictive, impulsive, and compulsive behaviors.

A wide range of neuroscientific studies have revealed that dopaminergic system plays a key role in these neural mechanisms involving reward and behaviors associated with obesity^1^ with a wide range of neural mechanisms. First, the psychological measure of condition of the diminished capacity for enjoying the reward is associated with a deficiency of dopamine receptor (particularly that of D2)^5^, and the sensitivity to reward is associated with dopamine availability or the sensitivity of dopamine pathway^6^. Greater responsivity in reward processing regions such as the caudate, putamen, and orbitofrontal cortex (OFC) and other networks in response to palatable food cues is associated with an increased risk for obesity^7^. Individuals with mild obesity show greater responsivity in these regions to palatable food cues compared with lean controls^8^. Exposure to food cues increases DA in the striatum and this response is associated with the desire to eat the food^9^. The involvement of dopamine in food reward is associated with “wanting” food, and this effect likely involves the dorsal striatum^3^. Mice that do not synthesize dopamine die of starvation due to lack of motivation to eat, whereas restoring dopamine neurotransmission in the dorsal striatum rescues these animals^10^. Neuroimaging studies showed an availability of dopamine receptor D2 is positively correlated with BMI in the striatal regions among individuals with mild obesity and without obesity^11^; however, this association was negative among individuals with morbid obesity (BMI > 40)^1^. Further, a wide range of previous studies have also investigated the associations of BMI with regional gray matter volume, cortical thickness, and white matter structural property (integrity) measured by fractional anisotropy of diffusion tensor imaging (DTI) as well as mean diffusivity (MD) measures of DTI in white matter areas^12,13^. Further, although obesity is suggested to downregulate dopaminergic function, hedonic eating is known to temporally increase dopamine release and suppress anxiety and depression, whereas stress, anxiety, and depression are suggested to lead to hedonic eating^14^.

So far, the associations between microstructural properties (MD) of the dopaminergic system and BMI have not been investigated. Previous studies have examined the association between obesity and MD in the white matter of the brain in a relatively small sample, but reported inconsistent results. Some studies reported an increase in MD, while other studies reported a decrease or no difference in MD (for review, see ref^15^). As summarized in our previous study^16^, the MD derived from DTI is considered a reflection of water diffusivity^17^. Greater tissue density, due to greater numbers of cellular structures, may prevent the free diffusion of water molecules, thereby decreasing the MD value^18–20^. As we reviewed previously^21^, MD in areas of the dopaminergic system (MDDS), particularly in subcortical areas, such as the putamen, caudate, and globus pallidus, is associated with several conditions related to alterations of the dopaminergic system. MD in areas of the dopaminergic system, such as the caudate and putamen, negatively correlated with dopamine synthesis capacity, as measured by positron emission tomography (partial correlation coefficient ≒ 0.7)^22^. MDDS has been shown to be a more sensitive index to detect the pathology of the dopaminergic system (Parkinson’s disease) than other magnetic resonance imaging (MRI) measures and positron emission tomography (PET)^23,24^. MDDS is negatively correlated with extraversion and novelty seeking, which have been associated with the dopaminergic activity^21^. In addition, MD and MDDS have been shown to be sensitive detectors of neural plasticity, including very rapid changes and changes caused by pharmacological and cognitive interventions related to dopamine^19,25,26^.

Because dopamine receptor availability is strongly associated with MDDS, we reasoned that MDDS would be negatively correlated with BMI among adults with mild obesity and without obesity.

In this study, we aimed to test these hypotheses and investigate the associations of BMI with MD, particularly MDDS, in normal young adults with mild obesity and without obesity (BMI < 40).

## Methods

### Subjects

Overall, 435 healthy, right-handed individuals (266 males and 169 females) participated in this study. The mean age of subjects was 20.8 years (standard deviation [SD], 1.6). All subjects had normal vision and none had a history of neurological or psychiatric illness. Handedness was evaluated using the Edinburgh Handedness Inventory^27^. All subjects were university students, postgraduates, or university graduates of less than one year’s standing. For further subject information, see Supplemental Methods. For limitations of this study related to subject characteristics, see Supplemental Discussion. Written informed consent was obtained from all participants. For nonadult subjects, written informed consent was also obtained from their parents (guardians)(In Japan, by law, those who are 20 years of age or older are considered to be adults. However, in the near future, those who are 18 years of age or older will be considered adults). The study was approved by the Ethics Committee of Tohoku University.

### BMI

We calculated the BMI of each participant by dividing weight in kilograms by the square of height in meters. Height was measured by a stadiometer and body weight by an electronic scale.

### Image acquisition

MRI data were acquired using a 3 T Philips Achieva scanner. Diffusion-weighted data were acquired using a spin-echo EPI sequence (TR = 10,293 ms, TE = 55 ms, FOV = 22.4 cm, 2 × 2 × 2 mm^3^ voxels, 60 slices, SENSE reduction factor = 2, number of acquisitions = 1). The diffusion weighting was isotropically distributed along 32 directions (*b* value = 1,000 s/mm^2^). Additionally, 3 images with no diffusion weighting (*b* value = 0 s/mm^2^, b = 0 images) were acquired using a spin-echo EPI sequence (TR = 10,293 ms, TE = 55 ms, FOV = 22.4 cm, 2 × 2 × 2 mm^3^ voxels, 60 slices). Further, there are acquisitions for phase correction and signal stabilization. These were not used as reconstructed images. FA and MD maps were calculated from the images using a commercially available diffusion tensor analysis package on an MR console which is provided by Philips (Eindhoven, the Netherlands). In this package calculations were performed according to a previously proposed method^28^. The FA and MD maps generated by this method showed results consistent with those of previous studies^29^. The descriptions from this subsection are mostly reproduced from our previous study using the exact same methods^30^. Apparent motion-induced artifacts were checked by visual inspection, and none were removed.

### Preprocessing of structural data

Preprocessing of the structural data was performed using Statistical Parametric Mapping software (SPM8; Wellcome Department of Cognitive Neurology, London, UK) implemented in Matlab (Mathworks Inc., Natick, MA, USA). The descriptions from this subsection are mostly reproduced from our previous study^31^, which used the exact same methods. First, using a previously validated, modified version of the diffeomorphic anatomical registration through exponentiated lie algebra (DARTEL)-based registration process which utilizes the information of both FA images and MD images (but not T1 weighted structural images), we segmented the FA images and MD images of the subjects. Then, using the diffeomorphic anatomical registration through exponentiated lie algebra registration process implemented in SPM8, (1) raw FA images, (2) raw MD images, (3) the regional gray matter density map [rGMD map], (4) the regional white matter density map [rWMD map], and (5) the regional cerebral spinal fluid (CSF) density map [rGMD map] from the second new segmentation process were normalized to yield images with 1.5 × 1.5 × 1.5 mm^3^ voxels. Subsequently, from the normalized images of the (a) MD map, (b) rGMD map, (c) rWMD map, and (d) rCSFD map, areas that were not strongly likely to be gray matter or white matter in our custom template (defined by “gray matter tissue probability + white matter tissue probability < 0.99” in the template) were removed to exclude the strong effects of CSF on MD. These images were then smoothed by convolving with an isotropic Gaussian kernel of 8-mm full width at half maximum. For additional details on these procedures, see Supplemental Methods.

### Whole-brain statistical analysis

We investigated associations of regional MD with individual differences in BMI. The statistical analyses of imaging data were performed with SPM8. Multiple regression analysis was performed, which included sex, age, TIV (which is calculated using voxel-based morphometry of T1-weighted structural images as described previously^32^, volume-level framewise displacement (FD) during the diffusion-weighted imaging scan (motion during the scan) and BMI. FD during the diffusion-weighted imaging scan was calculated based on the method used by Power et al.^33^ from the obtained all diffusion weighted images using SPM8’s extension software Data Processing Assistant for Resting-State fMRI (DPARSF) [part of the toolbox for Data Processing and Analysis of Brain Imaging (DPABI) (https://rfmri.org/dpabi)^34^].

The analyses were limited to the gray + white matter mask, which was created as described above.

A multiple comparison correction was performed using threshold-free cluster enhancement (TFCE)^35^ with randomized (5,000 permutations) nonparametric testing using the TFCE toolbox (https://dbm.neuro.uni-jena.de/tfce/). We applied a threshold of FWE corrected at *P* < 0.025 [0.05/2, Bonferroni correction of 2 contrasts (positive, negative relationship)].

### Region of interest (ROI) analyses of associations between MD and BMI

After identifying associations of MDDS with BMI (see “Results”), we employed ROI approaches to determine the associations between MD correlates of BMI (MDDS) and BMI after correcting for effects of regional gray matter density (rGMD) of the ROIs. The ROIs included the right globus pallidus and right putamen. All ROIs were constructed using the WFU PickAtlas Tool (https://www.fmri.wfubmc.edu/cms/software#PickAtlas)^36,37^. The mask images of the ROIs were generated using the Brodmann area option in the PickAtlas Tool. Subsequently, the mean MD and rGMD values of these images were extracted from the aforementioned normalized images. Here, values were extracted only from areas with “gray matter tissue probability + white matter tissue probability > 0.999” in the custom template mentioned above. The ROIs were anatomically defined to avoid “double dipping procedures”^38^ and overfitting effects of the whole brain analyses^39^.

The associations of MD values of anatomical ROI of the right putamen and right globus pallidus with BMI were tested by multiple regression analyses using SPSS 22.0 (SPSS Inc., Chicago, IL). The dependent variable was the mean MD in one of these ROIs and the independent variables comprised BMI, age, sex, TIV, FD and the mean rGMD value of the corresponding ROIs.

Among all ROI analyses, results with a threshold of *P* < 0.05 were considered to be statistically significant after correcting for the false discovery rate (FDR) using the classical one stage method^40^, were considered as statistically significant.

## Results

### Behavioral results

**Figure 1:**
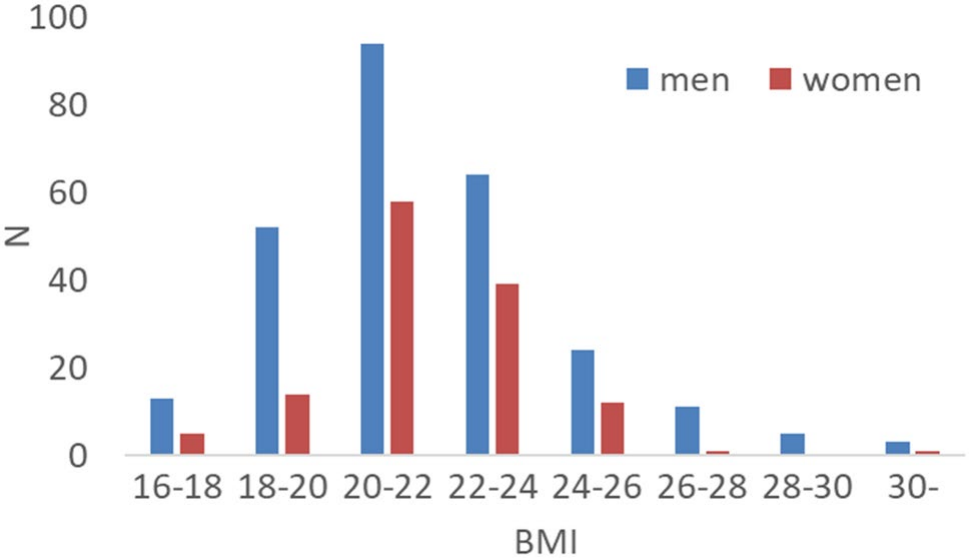
Distributions of BMI in male and female participants. The distribution in male and female was presented separately, as same BMI values have different meaning in two sexes.

Figure 1 presents the distribution of BMIs, and Table 1 shows the average results (and SDs) of the variables analyzed in this study sample for men and women. The proportion of participants who were classified as overweight according to the Asian standards^41^ (BMI: 23–25) was 16.5% (44/266) for males and 10.7% (18/169) for females. The proportion of participants who were classified as obese according to the Asian standards (BMI ≧ 25), but were classified as overweight according to the Western standards (BMI: 25–30) was 8.6% (23/266) for males and 5.3% (9/169) for females. The proportion of participants who were classified as obese according to the Western standards (BMI ≧ 30) was 1.1% (3/266) for males and 0.6% (1/169) for females. Mean and SD values of BMI were 21.75 ± 2.61 in males and 21.16 ± 2.15 in females. Although the sample included both males and females, we did not investigate sex differences in the neural correlates of BMI because of the limited numbers of participants.

**Table 1:** Mean and SD of the variables used in the study in males and females.

Measure	Males		Females	
	Mean	SD	Mean	SD
BMI (M:266, F:169)	21.75	2.61	21.16	2.15
Age (M:266, F:169)	20.81	1.74	20.66	1.46
TIV	1609.26	119.98	1,427.6	87.49
Mean framewise displacement of diffusion images	0.539	0.115	0.512	0.090

*TIV* total intracranial volume.

### Whole-brain analyses of correlations between BMI and MD

**Figure 2:**
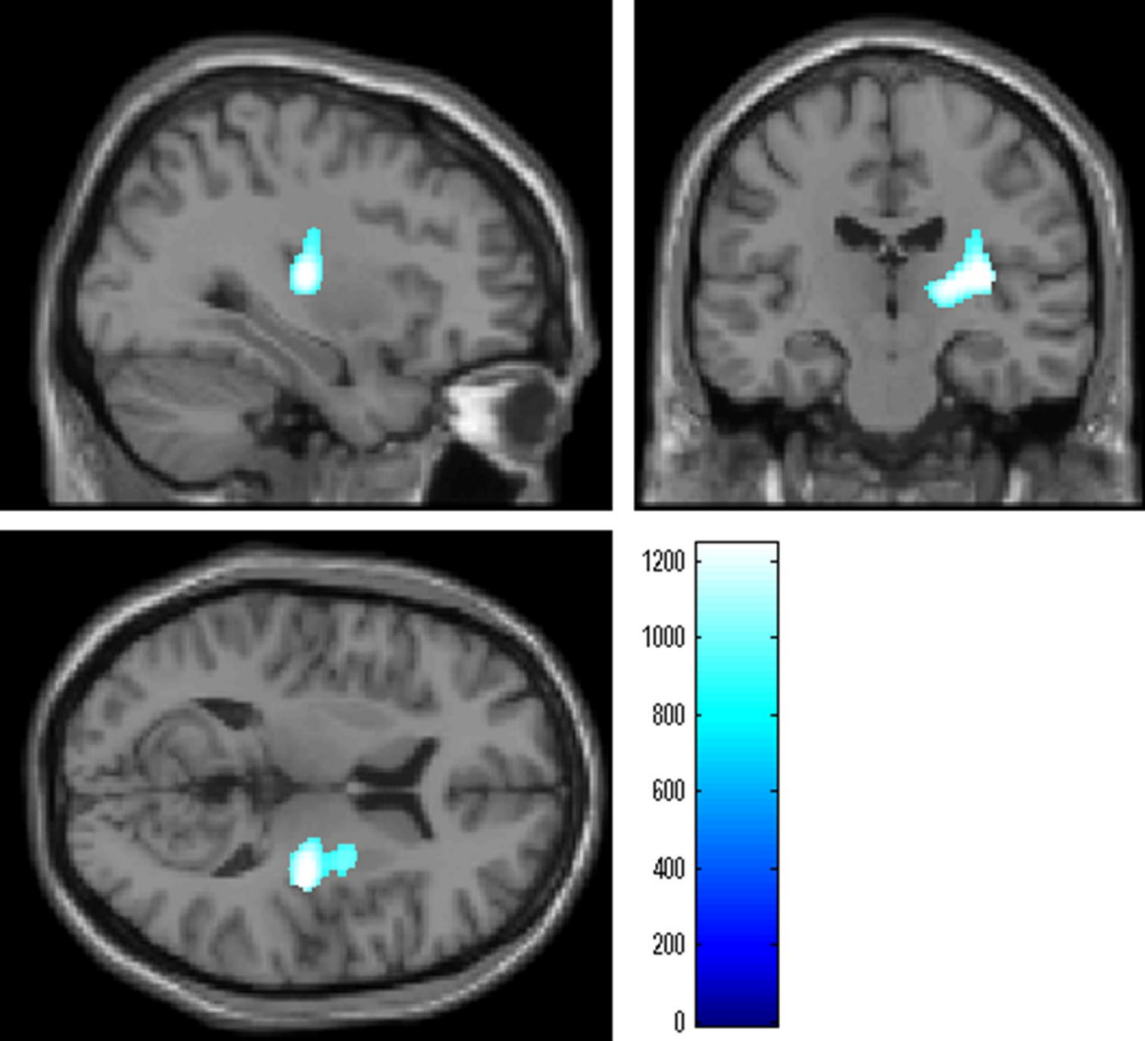
Regions with significant negative correlations between MD and BMI*.* The results shown were obtained using a threshold of threshold-free cluster enhancement (TFCE), *P* < 0.05 based on 5,000 permutations for the visualization purpose. Regions with significant correlations are projected onto a “single subject” T1 image from SPM8. The color represents the strength of the TFCE value. Regions with significant negative correlations were observed in the anatomical cluster spread around the right putamen, right globus pallidus, and right posterior insula.

Whole-brain multiple regression analysis, after controlling for sex, age, and TIV, showed that BMI was significantly and negatively correlated with MD in the anatomical cluster spread around the right globus pallidus, right putamen, and right posterior insula [Fig. 2, x, y, z = 33, − 18, 10.5, TFCE value = 1,241.9, *P* = 0.016, corrected for multiple comparison (FWE, permutation using TFCE value), 379 voxels below the threshold of *P* < 0.025, corrected]. There were no other significant relationships. These analyses did not consider the effects of rGMD differences on MD because we used nonparametric statistical tests that could not consider these effects. However, we carefully removed the CSF in the preprocessing stages, and the following ROI analyses that considered these effects yielded similar results.

### Region of interest (ROI) analyses of associations between MD and BMI

**Figure 3:**
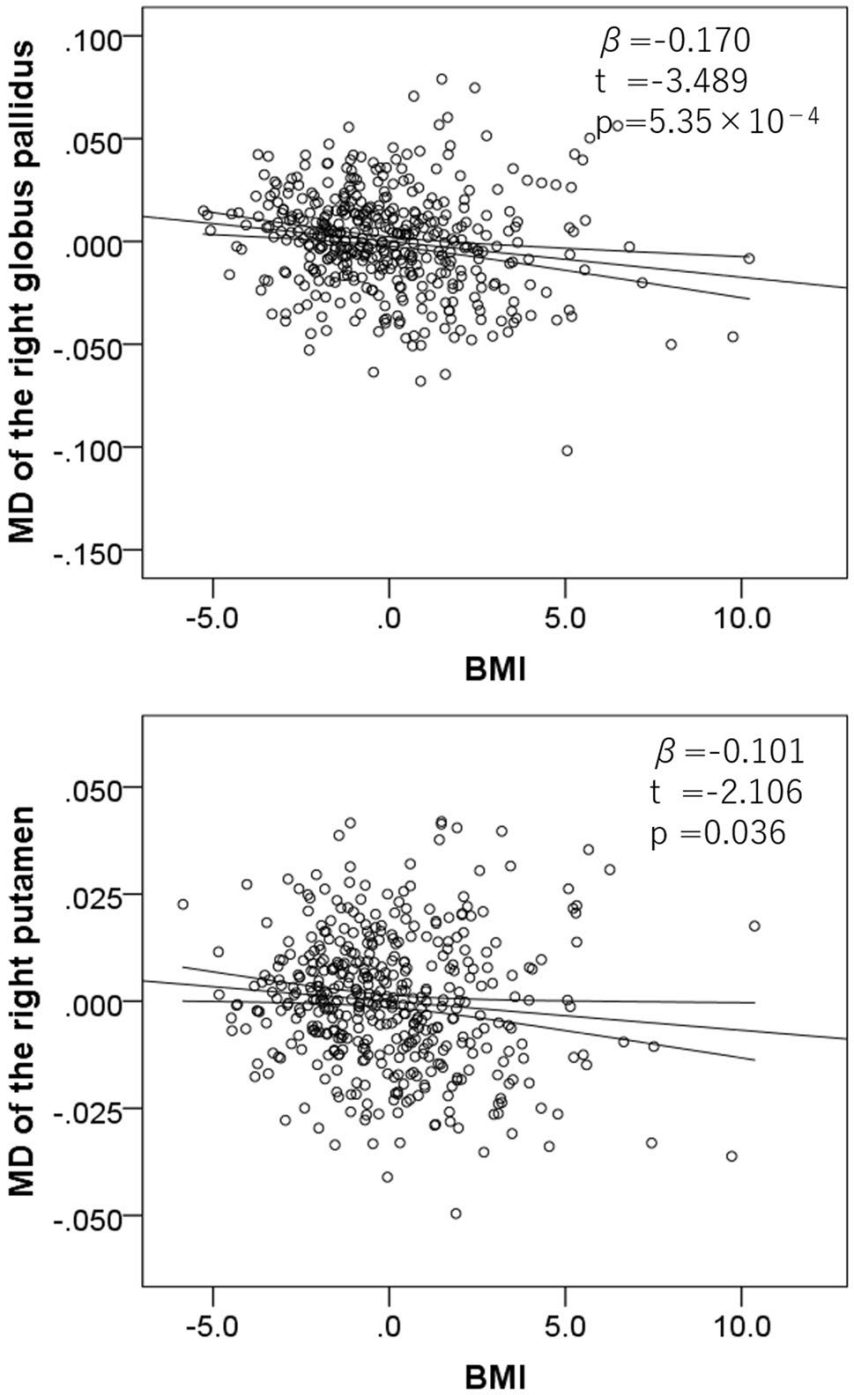
Associations of BMI with MD in the right globus pallidus and the right putamen. Residual plots with trend lines depicting the significant associations between residuals in the multiple regression analyses with MD of the right globus pallidus (**a**) or MD of the right putamen (**b**) as a dependent variable and BMI with other confounding factors as independent variables; 95% confidence intervals for the trend lines are shown.

We then investigated the associations between BMI and MD of the right globus pallidus and right putamen, regions that were anatomically defined after correcting for the effects of ROI rGMD as well as age, sex, FD, and TIV. The results showed that BMI significantly and negatively correlated with MD of the right globus pallidus and the right putamen (Fig. 3, *P* < 0.05, corrected for multiple comparisons).

For all of these statistical values, see Table 2.

**Table 2:** Multiple regression analyses for associations of BMI with regional MD.

Dependent variable	Right globus pallidus	Right putamen
Covariates	Age, sex, TIV, FD, rGMD of the area	Age, sex, TIV, FD rGMD of the area
	[β, *t P *(*uncorrected*), *P *(*FDR*)]	[β, *t P *(*uncorrected*), *P (FDR*)]
BMI	(− 0.170, − 3.489, 5.35 × 10^−4^, 0.001)*	(− 0.101, − 2.106, 0.036, 0.036)*

Upper left: beta value, upper right: *t*-value, lower left: uncorrected *P* value, lower right: *P* value corrected for FDR.

*FDR* false discovery rate.

## Discussion

In this study, we investigated the associations between BMI and MD. Particularly, we newly examined the association of BMI with subcortical MD, particularly MDDS. Our novel finding is that BMI is significantly and negatively associated with MD in the areas of the right globus pallidus, right putamen, and right posterior insula. This result is congruent with our hypothesis that BMI is associated with MDDS. The observed findings were not explained by differences in regional gray matter density since the ROI analyses correcting for effects of rGMD also identified the same findings. The sample size of this study is smaller than in some of our previous studies^42^ because we did not gather the measured (instead of self-reported) height and weight from all project participants. However, the supplemental ROI analyses, using the independent sample of 754 subjects in our previous study^43^ for whom self-reported height and weight data was available, replicated the significant associations between greater BMI and lower MD in the right putamen and the right globus pallidus (See Supplemental Methods and Supplemental Results).

Our findings suggest that MD in areas related to the dopaminergic system is associated with BMI. Each function of these regions may contribute to the motivational state. Dopaminergic neurons in the nigrostriatal system project into the putamen from the substantia nigra^44^, an area with critical functions in reward and motivation^45^. The globus pallidus is connected to the putamen and receives a dopaminergic input from the substantia nigra^46,47^; thus, it is an important part of the circuit of the dopaminergic system. The globus pallidus has been shown to play a central role in prediction of reward using a number of methods^48^. Thus, these regions may be associated with the sensitivity to reward, which is supposed to lead to the increase in eating behaviors and BMI through these cognitive and neural mechanisms.

In this study, we advanced our understanding of the associations of BMI with property of the dopaminergic system. It has been shown that among the adults with mild obesity and without obesity, BMI is positively correlated with the availability of D2 receptors^11^. Because dopamine synthesis capacity is known to strongly and negatively correlated with MDDS^22^, the present finding of the negative correlation between BMI and MDDS is in line with this previous study^11^.

The physiological mechanisms for the associations of MDDS with BMI are not clear; however, we suggest a possible speculative mechanism from the previously suggested notion. As described in the Introduction, it has been suggested that like individuals with drug addiction, individuals with obesity have lower reward sensitivity that arises from a primary deficiency of dopamine receptors (the reward deficiency hypothesis), resulting in a compensatory overconsumption of the reward^4^. An alternative view with experimental support is that (1) the sensitivity to reward conferred by the increased dopamine receptor D2 in the striatum leads to increased hedonic eating behaviors in adults with mild obesity and without obesity, resulting in an increase in the BMI and obesity, (2) but this association is reversed in the sample with morbid obesity because of downregulation of D2 receptors. Further, MDDS is strongly and negatively correlated with the availability of D2 receptors^22^.

One limitation of the present study is that the sampling was limited; it included only young, healthy, and well educated adults. The limited sampling is a common limitation of many studies using a college cohort^49^. Unlike a previous study investigating the association between dopamine receptor availability and BMI^1^, the current study did not include individuals with high obesity (BMI > 40) as well as subjects of anorexia nervosa, although, in Japan obesity is defined as BMI > 25, and not 30 unlike in western countries^50^ and the present sample include some samples with obesity. As a result, although previous studies suggested a non-linear association between obesity and mood or dopaminergic activity, we could only investigate the associations among the sample with subjects without severe obesity (BMI < 35)^6^. In addition, previous studies revealed ethnic differences in the effects of BMI on mood states, and young white females showed stronger associations between depressed mood and obesity than young Hispanic and black females^51^. A previous study revealed sex differences in the associations among BMI, mood, and brain structures^13,52^, but the present study could not investigate these differences because of the small sample size. Future studies are needed to confirm whether the present findings can be generalized to the broader population and possible sex differences of associations of BMI with MD.

In conclusion, among subjects without severe obesity, lower MDDS is associated with greater BMI. These results suggest that, among the adults with mild obesity and without obesity, the dopaminergic system is associated with BMI. Our results are in line with the previously suggested notion that hyperfunction of the dopaminergic system is associated with a tendency of obesity.

### Ethical statement

All procedures performed in studies involving human participants were in accordance with the ethical standards of the institutional and/or national research committee and with the 1964 Helsinki declaration and its later amendments or comparable ethical standards.

### Ethical approval

This study was approved by the Ethics Committee of Tohoku University.

### Informed consent

Informed consent was obtained from all individual participants included in the study.

## Supplementary information

Supplementary Information.
