# Supplementary material for: The associations of BMI with mean diffusivity of basal ganglia among young adults with mild obesity and without obesity
Source: Sci Rep. 2020 Jul 28;10:12566. doi: 10.1038/s41598-020-69438-5 (PMC7387490; doi:10.1038/s41598-020-69438-5)
Supplement: Supplementary file 1 — Supplementary Information. [file 41598_2020_69438_MOESM1_ESM.pdf]

The associations of BMI with mean diffusivity of basal ganglia among young adults with mild obesity and without obesity

Hikaru Takeuchi[a], Yasuyuki Taki[a,b,c], Rui Nouchi[d,e,f], Ryoichi Yokoyama[g], Seishu Nakagawa[h,i], Kunio Iizuka[j], Kohei Sakaki[f], Tsuyoshi Araki[k], Takayuki Nozawa[l], Shigeyuki Ikeda[m], Susumu Yokota[a], Sugiko Hanawa[h], Magistro Daniele[n], Yuka Kotozaki[o], Yukako Sasaki[f], Kelssy H. dos S. Kawata[p], Ryuta Kawashima[a,d,f]

[a]*Division of Developmental Cognitive Neuroscience, Institute of Development, Aging and Cancer, Tohoku University, Sendai, Japan*

[b]*Division of Medical Neuroimaging Analysis, Department of Community Medical Supports, Tohoku Medical Megabank Organization, Tohoku University, Sendai, Japan*

[c]*Department of Radiology and Nuclear Medicine, Institute of Development, Aging and Cancer, Tohoku University, Sendai, Japan*

[d]*Creative Interdisciplinary Research Division, Frontier Research Institute for Interdisciplinary Science, Tohoku University, Sendai, Japan*

[e]*Human and Social Response Research Division, International Research Institute of Disaster Science, Tohoku University, Sendai, Japan*

[f]*Department of Advanced Brain Science, Institute of Development, Aging and Cancer, Tohoku University, Sendai, Japan*

[g] *School of Medicine, Kobe University, Kobe, Japan*

[h]*Department of Human Brain Science, Institute of Development, Aging and Cancer, Tohoku University, Sendai, Japan*

[i] *Division of Psychiatry, Tohoku Medical and Pharmaceutical University, Sendai, Japan*

[j] *Division of Psychiatry, Tohoku Medical and Pharmaceutical University, Sendai, Japan*

[k] *ADVANTAGE Risk Management Co., Ltd.*

[l] *Collaborative Research Center for Happiness Co-Creation Society through Intelligent Communications, Tokyo Institute of Technology, Tokyo, Japan*

[m] *Department of Ubiquitous Sensing, Institute of Development, Aging and Cancer, Tohoku University, Sendai, Japan*

[n]*National Centre for Sport and Exercise Medicine (NCSEM), The NIHR Leicester-Loughborough Diet, Lifestyle and Physical Activity Biomedical Research Unit, School of Sport, Exercise, and Health Sciences, Loughborough University, England*

[o]*Division of Clinical research, Medical-Industry Translational Research Center, Fukushima Medical University School of Medicine, Fukushima, Japan*

[p] *Center for Evolutionary Cognitive Sciences, University of Tokyo, Tokyo, Japan*

Corresponding author:

Hikaru Takeuchi

Division of Developmental Cognitive Neuroscience, IDAC, Tohoku University

4-1 Seiryo-cho, Aoba-ku, Sendai 980-8575, Japan

Tel/Fax: +81-22-717-7988

E-mail: takehi@idac.tohoku.ac.jp

Short title: BMI, mean diffusivity and motivational state

Keywords: BMI, obesity, mood state, mean diffusivity, dopaminergic system, motivation

Supplemental online material

Supplemental Methods

Subjects. The present study, which is a part of an ongoing project to investigate the association between brain imaging, cognitive function, and aging, included BMI measures and imaging data from 435 healthy, right-handed individuals (266 males and 169 females). The mean age of the subjects was 20.8 years [standard deviation (SD), 1.6; age range, 18−27 years old]. The following descriptions were mostly reproduced from another study of ours from the same project using the exactly same methods regarding these issues (Hikaru Takeuchi et al., 2015). Some of the subjects who took part in this study also became subjects of our intervention studies (psychological data and imaging data recorded before the intervention were used in this study) (Maruyama et al., 2018). Psychological tests and MRI scans not described in this study were performed together with those described in this study. This study was approved by the Ethics Committee of Tohoku University.

Subjects were instructed to get sufficient sleep, maintain their conditions, eat sufficient breakfast, and to consume their normal amounts of caffeinated foods and drinks in the day of cognitive tests and MRI scans. In addition, subjects were instructed to avoid alcohol the night before the assessment.

Preprocessing of imaging data

Preprocessing and analysis of functional activation data were performed using SPM8 implemented in Matlab. Most of the following descriptions were reproduced

from our previous study using the similar methods (Hikaru Takeuchi et al., 2016). First, the skull in the mean b = 0 image of each participant was stripped as described previously (H. Takeuchi et al., 2010); using the resulting image, diffusion images were linearly aligned to the skull-stripped b = 0 image template created previously (H. Takeuchi et al., 2010) to assist with the following procedures.

Subsequently, using a previously validated, two-step, new segmentation algorithm of diffusion images and the previously validated diffeomorphic anatomical registration through exponentiated lie algebra (DARTEL)-based registration process that utilized the information of the FA signal distribution within the white matter tissue (for details, see Hikaru Takeuchi et al., 2013), all images, including gray matter segments [regional gray matter density (rGMD) map], white matter segments [regional white matter density (rWMD) map], and cerebrospinal fluid (CSF) segments [regional CSF density (rCSFD) map] of diffusion images were normalized. The voxel size of these normalized images was $1.5 \times 1.5 \times 1.5$ mm^3. In these processes, we used the template for the DARTEL process that we created in our previous study from subjects that participated in the same project (for details, see Hikaru Takeuchi et al., 2013).

Next, we created average images of normalized rGMD and rWMD images of all subjects whose diffusion imaging data were obtained in the pre-experiment.

Subsequently, for the analyses of MD images from the normalized images of the (a) MD, (b) rGMD, and (c) rWMD maps, we created images where areas that were not strongly likely to be gray or white matter in our averaged normalized rGMD and rWMD images (defined by "gray matter tissue probability + white matter tissue probability < 0.99") were removed (to exclude the strong effects of CSF on MD throughout analyses). These images were then smoothed (8 mm full-width half-maximum) and carried through to the second-level analyses of MD.

We did not use T1 weighted structural images for normalization and calculation of GMC and WMC maps for correction. This is because T1 weighted structural images and EPI images have apparent differences due to the distortion caused by 3T MRI and, simply, it is apparently not suited for the accurate and precise segmentation and normalization images of MD maps.

Supplemental replication ROI analyses of the associations between BMI calculated from self-reported data and MD using the independent sample.

In addition, we conducted supplemental analyses to investigate whether the associations between BMI, and the MD of the right putamen and right globus pallidus were replicated in the independent sample for whom self-reported height and weight data was available,. In this analysis, we used the sample from our previous study, which did not have measured

height and weight data, but had self-reported height and weight data and the same parameters for diffusion weighted imaging. There were 754 subjects (421 males, 332 females) with all the necessary data. The subjects' mean age was 20.8 years (standard deviation [SD], 2.0). The mean and SD values of BMI were 20.99 ± 2.35 in males and 20.31 ± 1.85 in females. All the preprocessing and analytic procedures were conducted using the same methods used in the preprocessing and analyses of the ROI in the main text.

Supplemental Results.

Supplemental replication ROI analyses of the associations between BMI calculated from self-reported data and MD using the independent sample

We investigated the associations between BMI, calculated from self-reported weight and height data, and the MD of the right globus pallidus and right putamen, regions that were anatomically defined after correcting for the effects of each ROI's rGMD, as well as age, sex, FD, and TIV. The results showed that BMI significantly and negatively correlated with the MD of the right globus pallidus [$\beta = -0.177$, $t = -4.905$, P (uncorrected) = 0.000001, P (corrected for FDR among 2 supplemental ROI analyses) = 0.000002] and the right putamen [$\beta = -0.143$, $t = -3.951$, P (uncorrected) = 0.000085, P (corrected for FDR among 2 supplemental ROI analyses) = 0.000085]. These results replicate the significant associations of greater BMI with lower MD in the right globus pallidus, and right putamen which were observed in the main text, using the

independent sample and BMI based on the self-reported weight and height data.

References

Maruyama, T., Takeuchi, H., Taki, Y., Motoki, K., Jeong, H., Kotozaki, Y., Shinada, T., Nakagawa, S., Nouchi, R., Iizuka, K., Yokoyama, R., Yamamoto, Y., Hanawa, S., Araki, T., Sakaki, K., Sasaki, Y., Magistro, D., & Kawashima, R. (2018). Effects of time-compressed speech training on multiple functional and structural neural mechanisms involving the left superior temporal gyrus. *Neural Plasticity, 2018*, Article ID 6574178, 6574112 pages.

Takeuchi, H., Taki, Y., Hashizume, H., Asano, K., Asano, M., Sassa, Y., Yokota, S., Kotozaki, Y., Nouchi, R., & Kawashima, R. (2016). Impact of videogame play on the brain's microstructural properties: Cross-sectional and longitudinal analyses. *Molecular Psychiatry, 21*, 1781-1789.

Takeuchi, H., Taki, Y., Nouchi, R., Sekiguchi, A., Hashizume, H., Sassa, Y., Kotozaki, Y., Miyauchi, C. M., Yokoyama, R., Iizuka, K., Seishu, N., Tomomi, N., Kunitoki, K., & Kawashima, R. (2015). Degree centrality and fractional amplitude of low-frequency oscillations associated with Stroop interference. *Neuroimage, 119*(1), 197-209.

Takeuchi, H., Taki, Y., Sassa, Y., Hashizume, H., Sekiguchi, A., Fukushima, A., & Kawashima, R. (2010). White matter structures associated with creativity: Evidence from diffusion tensor imaging. *Neuroimage, 51*(1), 11-18.

Takeuchi, H., Taki, Y., Thyreau, B., Sassa, Y., Hashizume, H., Sekiguchi, A., Nagase, T., Nouchi, R., Fukushima, A., & Kawashima, R. (2013). White matter structures associated with empathizing and systemizing in young adults. *Neuroimage,*

77(15), 222-236.
